# Dimeric and Trimeric Fusion Proteins Generated with Fimbrial Adhesins of Uropathogenic *Escherichia coli*

**DOI:** 10.3389/fcimb.2016.00135

**Published:** 2016-10-31

**Authors:** Víctor M. Luna-Pineda, Juan Pablo Reyes-Grajeda, Ariadnna Cruz-Córdova, Zeus Saldaña-Ahuactzi, Sara A. Ochoa, Carmen Maldonado-Bernal, Vicenta Cázares-Domínguez, Leticia Moreno-Fierros, José Arellano-Galindo, Rigoberto Hernández-Castro, Juan Xicohtencatl-Cortes

**Affiliations:** ^1^Laboratorio de Investigación en Bacteriología Intestinal, Hospital Infantil de México “Federico Gómez”Ciudad de México, Mexico; ^2^Instituto de Fisiología Celular, Universidad Nacional Autónoma de MéxicoCiudad de México, Mexico; ^3^Instituto Nacional de Medicina GenómicaCiudad de México, Mexico; ^4^Laboratorio de Investigación de Inmunología y Proteómica, Hospital Infantil de México “Federico Gómez”, Dirección De InvestigaciónCiudad de México, Mexico; ^5^Unidad de Biomedicina, Laboratorio de Inmunidad en Mucosas, Facultad de Estudios Superiores Iztacala, Universidad Nacional Autónoma de MéxicoTlalnepantla, Mexico; ^6^Departamento de Infectología, Hospital Infantil de México “Federico Gómez”Ciudad de México, Mexico; ^7^Departamento de Ecología de Agentes Patógenos, Hospital General “Dr. Manuel Gea González”Ciudad de México, Mexico

**Keywords:** UPEC, fimbrial adhesin, fusion protein, UTIs, cytokines

## Abstract

Urinary tract infections (UTIs) are associated with high rates of morbidity and mortality worldwide, and uropathogenic *Escherichia coli* (UPEC) is the main etiologic agent. Fimbriae assembled on the bacterial surface are essential for adhesion to the urinary tract epithelium. In this study, the FimH, CsgA, and PapG adhesins were fused to generate biomolecules for use as potential target vaccines against UTIs. The fusion protein design was generated using bioinformatics tools, and template fusion gene sequences were synthesized by GenScript in the following order *fimH*-*csgA*-*papG*-*fimH*-*csgA* (*fcpfc*) linked to the nucleotide sequence encoding the [EAAAK]_5_ peptide. Monomeric (*fimH, csgA*, and *papG*), dimeric (*fimH-csgA*), and trimeric (*fimH*-*csgA*-*papG*) genes were cloned into the pLATE31 expression vector and generated products of 1040, 539, 1139, 1442, and 2444 bp, respectively. Fusion protein expression in BL21 *E. coli* was induced with 1 mM IPTG, and His-tagged proteins were purified under denaturing conditions and refolded by dialysis using C-buffer. Coomassie blue-stained SDS-PAGE gels and Western blot analysis revealed bands of 29.5, 11.9, 33.9, 44.9, and 82.1 kDa, corresponding to FimH, CsgA, PapG, FC, and FCP proteins, respectively. Mass spectrometry analysis by MALDI-TOF/TOF revealed specific peptides that confirmed the fusion protein structures. Dynamic light scattering analysis revealed the polydispersed state of the fusion proteins. FimH, CsgA, and PapG stimulated the release of 372–398 pg/mL IL-6; interestingly, FC and FCP stimulated the release of 464.79 pg/mL (*p* ≤ 0.018) and 521.24 pg/mL (*p* ≤ 0.002) IL-6, respectively. In addition, FC and FCP stimulated the release of 398.52 pg/mL (*p* ≤ 0.001) and 450.40 pg/mL (*p* ≤ 0.002) IL-8, respectively. High levels of IgA and IgG antibodies in human sera reacted against the fusion proteins, and under identical conditions, low levels of IgA and IgG antibodies were detected in human urine. Rabbit polyclonal antibodies generated against FimH, CsgA, PapG, FC, and FCP blocked the adhesion of *E. coli* strain CFT073 to HTB5 bladder cells. In conclusion, the FC and FCP proteins were highly stable, demonstrated antigenic properties, and induced cytokine release (IL-6 and IL-8); furthermore, antibodies generated against these proteins showed protection against bacterial adhesion.

## Introduction

Urinary tract infections (UTIs) are associated with high rates of morbidity and mortality worldwide (Foxman, 2010; Flores-Mireles et al., 2015; Kumar et al., 2015). In Mexico, UTIs are a public health problem and are the third leading cause of morbidity, with ~4 million cases per year (Secretaria de Salud, 2009). Populations with a high risk of acquiring UTIs include newborns, preschool girls, women who are sexually active, and both sexes at advanced ages (Foxman, 2002, 2014).

Uropathogenic *Escherichia coli* (UPEC) is the primary etiologic agent responsible for UTIs, which are classified according to the site of infection: urine (asymptomatic bacteriuria), bladder (cystitis), kidney (pyelonephritis), and blood (urosepsis and bacteremia; Foxman, 2002). The pathogenic mechanism of UPEC begins with adherence via fimbrial adhesins (FimH, PapG, SfaS, and FocH), which are assembled on the distal tip of type 1, P, S, and F1C fimbriae, respectively. Additionally, CsgA (Curli fimbriae) and DrA (Dr fimbriae) proteins have been implicated in epithelial cell adhesion (Antão et al., 2009). These adhesins interact with different receptors (α-D-mannosylated proteins, glycosphingolipids, neuraminic acid, lactosylceramide, decay accelerating factor, and matrix proteins) located on the membrane of cells of the urinary tract (Antão et al., 2009; Lüthje and Brauner, 2014). The FimH adhesin of type 1 fimbriae interacts with uroplakin proteins in the bladder, resulting in an invasion process that allows UPEC to avoid urine flow, antibodies, bactericidal molecules, and antibiotic activity in the urinary tract (Mulvey et al., 1998, 2000; Zhou et al., 2001). UPEC produces biofilm-like structures called intracellular-bacterial communities (IBCs) within the cytoplasm of urothelial cells, conferring protection to the bacteria and facilitating their egress to promote a new cycle of infection through bladder cell lysis (Scott et al., 2015). During infection cycles, UPEC enter a quiescent state for long periods of time, and this quiescence constitutes a mechanism for bacterial persistence (Leatham-Jensen et al., 2016). UPEC then exit the quiescent state by promoting exocytosis from bladder cells and infecting new cells, resulting in recurrent UTIs (rUTIs, Leatham-Jensen et al., 2016). Three percent of women with three or more rUTIs annually are at risk for developing pyelonephritis and urosepsis (Foxman, 2002, 2010). UTIs are typically treated with several broad-spectrum antibiotics (ampicillin, trimethoprim/sulfamethoxazole, fluoroquinolones, and cephalosporin), resulting in increased resistance rates among clinical UPEC strains. This resistance complicates treatment, increases costs, and decreases the efficiency of antibiotics against infection (Biedenbach et al., 2016). The indiscriminate use of antibiotics modifies the commensal microbiota of patients and generates secondary infections (candida-vaginal and gastrointestinal infections) during and after prophylactic treatment (Flores-Mireles et al., 2015).

The FimH adhesin of UPEC type 1 fimbriae has been used as a biomolecule to induce protection in murine models (Langermann et al., 1997, 2000; Langermann and Ballou, 2001). During infection, type 1 fimbrial expression is regulated by environmental conditions (temperature, osmolality, pH, and nutrients) as well as the specific anatomic site of infection in the urinary tract (bladder, ureters, and kidney). These conditions also dictate the expression of other fimbriae that promote urinary tract colonization by UPEC (Snyder et al., 2005; Greene et al., 2015).

P fimbriae of UPEC have been associated with colonization and inflammation in the kidneys (pyelonephritis) through binding of PapG adhesion to Gal(α1-4)Gal-containing glycolipids and Toll-like receptor (TLR) 4 in kidney cells (Frendéus et al., 2001; Lane and Mobley, 2007). Curli fimbriae are primarily composed of CsgA protein monomers and are associated with cystitis, pyelonephritis, and bacteremia (Norinder et al., 2012; Hung et al., 2014; Lim et al., 2014). These fimbriae are widely distributed in clinical strains of UPEC and are finely regulated by a regulatory system of on-to-off and/or off-to-on switching (Snyder et al., 2005). Recombinant proteins generated using fusion technology to contain one or more antigens derived from diverse pathogens can induce immune and protective responses against UTIs in *in vivo* models (Asadi Karam et al., 2013; Habibi et al., 2015b). Vaccines designed based on fused proteins of UPEC clinical strains with high variability in their virulence factors might represent potential tools for preventing UTIs. The aim of this study was to generate recombinant fusion proteins (combination of FimH, PapG, and CsgA proteins) as viable biomolecules with vaccine properties to significantly reduce UTIs in Mexico.

## Materials and methods

### Primary, secondary, and tertiary structures

FimH, CsgA, and PapG protein sequences from the *E. coli* strain CFT073 were obtained from the NCBI database (http://www.ncbi.nlm.nih.gov/protein) under accession numbers AAN83822.1, AAN79779.1, and AAN82031.1, respectively. Signal peptide prediction was performed for each protein using the SignalP 4.1 server, and five repetitions of the EAAAK sequence were employed to fuse the FimH, CsgA, and PapG proteins based on the linker library construction proposed by Li et al. (2016).

In addition, the molecular weight, theoretical isoelectric point (pI), amino acid composition, estimated lifetime, aliphatic index, and grand average of hydropathicity (GRAVY) of the fusion proteins were determined using the ProtParam program of ExPASy (Wilkins et al., 1999). The Codon Adaptation Index (CAI) and guanine and cytosine (GC) content of the genes were determined using the OPTIMIZER program (Puigbò et al., 2007). The secondary structure of the fusion proteins was predicted using the GOR IV program (Sen et al., 2005). Fusion protein modeling was performed using the hierarchical modeling approach (I-TASSER) and visualized with PyMOL software (Yang et al., 2015). Tridimensional (3D) structures were refined and minimized with KoBaMIN (http://csb.stanford.edu/kobamin/) and VegaZZ (NAMD; Pedretti et al., 2004). The 3D models were then validated by Protein Structure Analysis (ProSA) to determine *Z*-scores and Ramachandran plots using PROCHECK (Laskowski et al., 1998; Wiederstein and Sippl, 2007). Merging of the 3D fusion proteins with the crystal structures of the mannose-binding domain FimH (Protein Data Bank; 1TR7) and lectin domain PapG (PDB; 1J8R) were calculated with the root mean square deviation (RMSD) using the TM-align program (Zhang and Skolnick, 2005).

### Epitope prediction and antigen presentation

Immune response was theoretically determined to establish variants of fusion proteins with enhanced ability to generate an immune response. The primary and secondary structures of the fusion proteins were employed to determine the lineal antigenic epitopes of B with the AbcPred server (http://www.imtech.res.in/raghava/abcpred/), and peptides with affinities for major histocompatibility complex (MHC) class II were identified with the NetMHCII program (http://www.cbs.dtu.dk/services/NetMHCII/). The 3D structures of the fusion proteins were employed to determine the conformational antigenic epitopes using the Discotope server (http://www.cbs.dtu.dk/services/DiscoTope/).

### Analysis and synthesis of a template fusion gene

The gene sequences of *fimH, csgA*, and *papG* from the *E. coli* strain CFT073 were obtained from GenBank through NCBI (http://www.ncbi.nlm.nih.gov/genbank/) under the accession numbers GQ487191.1, NC_004431.1, and AF447814.1, respectively. The conserved sequences of the *fimH, csgA*, and *papG* genes from various UPEC strains (UTI89, ABU83972, NA114, UPEC 26-1, CF-088, CF-468, isolates IA2, and AD110) were determined using the BLAST (http://blast.ncbi.nlm.nih.gov/Blast.cgi) and Clustal Omega (http://www.ebi.ac.uk/Tools/msa/clustalo/) programs. Consensus nucleotide sequences were fused with the appropriate codons from the sequence EAAAK to generate a template fusion gene. The sequence of the template fusion gene was optimized using the OPTIMIZER program (http://genomes.urv.es/OPTIMIZER/), and messenger RNA secondary structures of monomeric, dimeric, and trimeric genes were predicted using the Mfold program (http://unafold.rna.albany.edu/?q=mfold/RNA-Folding-Form). The optimized template fusion gene, containing a 5′ *BamH*I site and 3′ *Sac*I site, was synthesized by GenScript (Piscataway, NJ, USA) and cloned into the pUC57 vector to amplify and clone monomeric, dimeric, and trimeric genes.

### Cloning of monomeric and fusion genes into the pLATE expression vector

Primer designs for monomeric, dimeric, and trimeric genes from the synthetized template fusion gene were generated manually according to the aLICator LIC Cloning and Expression handbook (Thermo Fisher Scientific, Waltham, MA, USA) and synthesized by IDT Technologies (Coralville, Iowa, USA). Gene amplification was performed by polymerase chain reaction (PCR) using *Pfu* DNA polymerase. Conditions for amplification and cloning were obtained from the aLICator LIC Cloning and Expression handbook. The pLATE31 expression vector was used to clone the fusion genes and transformed by electroporation into BL21 (DE3) *E. coli*.

### Sequencing and GenBank submission of the fusion genes

Fusion genes in recombinant vectors were verified by next-generation sequencing on a NexSeq 500 system (Illumina, San Diego, CA, USA). Specific sequencing primers were obtained from the aLICator LIC Cloning and Expression Kit, and the gene sequences were analyzed by BLAST and submitted to GenBank at NCBI.

### Expression and purification of fusion proteins

A BL21 *E. coli* strain carrying the pLATE31 expression vector (fusion proteins) was plated on Luria Bertani (LB; Becton-Dickinson, Franklin Lakes, New Jersey, USA) agar and incubated for 16 h at 30°C. The putative transformant colonies were selected via the colony-blotting method using an anti-6X His (C-Term) HRP antibody (Abcam; Cambridge, MA, USA). Solubility assays were performed following the protocol described in the QIAexpressionist manual from Qiagen (Jacques-Schiesser-Str, Stockach, Germany). Fusion protein expression was performed in 500 mL of LB medium supplemented with 1 mM IPTG for 5 h at 37°C. The cell pellets were resuspended in phosphate buffer (10 mM K_2_HPO_4_, pH 7.4, 150 mM NaCl, and 1 mM EDTA) with phenylmethylsulfonyl fluoride (PMSF; Sigma-Aldrich Corp., St. Louis, MO, USA), lysed by sonication, and centrifuged at 26,116 g for 20 min. The supernatants were discarded, and the pellets were resuspended in denaturing buffer (8 M guanidine, 100 mM NaCl, and 100 mM K_2_HPO_4_, pH 8). After 3 days of incubation at room temperature, the lysates were centrifuged at 58,762 g for 20 min. The supernatants were incubated on a column containing nickel-nitrilotriacetic acid-agarose (Qiagen) at 4°C for 1 h, washed with A-buffer (8.5 M urea, 20 mM Tris, pH 7.5, 160 mM NaCl, and 20 mM imidazole) and eluted with B-buffer (8 M urea, 50 mM Na_2_HPO_4_, pH 8, 100 mM NaCl, and 500 mM imidazole). The collected proteins underwent refolding via dialysis using a urea gradient from 7 to 1 M in C-buffer (25 mM Tris, pH 7.5, 100 mM NaCl, and 0.5 mM EDTA); incubation was performed at 4°C for 24 h. Refolded fusion proteins were stored in C-buffer at −70°C for all assays.

### Characterization of fusion proteins

Fusion proteins were quantified according to the protocol for the 2D-Quant kit (GE Healthcare Bio-Sciences AB, Björkgatan, Uppsala, Sweden), separated by performing 14% sodium dodecyl sulfate polyacrylamide gel electrophoresis (SDS-PAGE), visualized by Coomassie staining and identified by mass spectroscopy using a 4800 MALDI TOF/TOF™ Analyzer (Applied Biosystems/MDS SCIEX, Waltham, MA, USA) after excision of the proteins spots. The CsgA protein was treated with 88% formic acid (Sigma-Aldrich Corp., St. Louis, MO, USA) prior to SDS-PAGE (Saldaña et al., 2009). Molecular weights were estimated using Image Lab software version 5.2 from Bio-Rad (Hercules, California, USA). The aggregation state of fusion proteins was determined by performing dynamic light scattering (DLS) using a Zetasizer Helix (Malvern Instruments Ltd, Grovewood Road, Worcestershire, United Kingdom). Fusion proteins with histidine tags were transferred onto polyvinylidene difluoride (PVDF) membranes and confirmed by performing Western blot assays using anti-6His (C-Terminal) HRP antibodies (Abcam; Cambridge, MA, USA) as described by Ledesma et al. (2010). The endotoxin (LPS) levels of the purified fusion proteins were determined using the Pierce™ LAL Chromogenic Endotoxin Quantitation kit according to the manufacturer's protocol (Thermo Fisher Scientific; Waltham, MA, USA). Additionally, fusion proteins were treated with 50 μg/mL polymyxin B (Sigma-Aldrich Corp., St. Louis, MO, USA) for 12 h at 4°C prior to bioactivity assays.

### Bioactivity assays using the fusion proteins

TLR2 and TLR4 expression in HTB5 bladder cells [American Type Culture Collection (ATCC), Manassas, VA, USA] was analyzed by flow cytometry using a human TLR2 fluorescein-conjugated antibody (R&D Systems, Inc., Minneapolis, USA) and a human TLR4/MD-2 complex phycoerythrin-conjugated antibody (Santa Cruz Biotechnology Inc., Texas, USA). Activation of TLR2 and TLR4 by the fusion proteins was assessed by quantifying the release of IL-6 and IL-8. HTB5 cells were cultured in 24 well-plates (Greiner, Germany) at a density of 10^5^ cells/well incubated with 1 mL of fresh Eagle's minimum essential medium (EMEM; ATCC® 30-2003™) supplemented with 10% fetal bovine serum (FBS) from Gibco (Thermo Fisher Scientific; Waltham, MA, USA). Cytokine induction in HTB5 cells was detected after 6 h of incubation with 10 μg/mL FimH, CsgA, PapG, FC, and FCP proteins by performing enzyme-linked immunosorbent assays (ELISAs) following the protocol established by BD Biosciences (San Jose, CA, USA). In addition, 100 ng/mL lipopolysaccharide (LPS; Sigma-Aldrich Corp., St. Louis, MO, USA) from *E. coli* 0111:B4 and 100 ng/mL lipoteichoic acid (LTA; Sigma-Aldrich Corp., St. Louis, MO, USA) from *S. aureus* were used as controls for the induction of TLR4 and TLR2, respectively.

### Antigenicity of fusion proteins

The study was approved by the Research (Dr. Onofre Muñoz Hernández), Ethics (Dr. Amparo Faure Fontenla), and Biosecurity (Dr. Herlinda Vera Hermosillo) Committees of Hospital Infantil de México Federico Gómez (HIMFG) under numbers HIM/2014/022 and HIM/2016/027. Physicians from the Infectology Department obtained permission and consent from patients to use the urine and serum samples employed in this study. Serum and urine samples were collected from 14 UTI patients (UP); samples from 14 healthy patients (HP) recruited from “Laboratorio Central del HIMFG” were used as negative controls. Patients were selected based on the following criteria: UTI symptoms, urine culture with ≥100,000 CFU/mL *E. coli*, leukocyte esterase and/or nitrites, and urothelial cells in urine. Urine samples were centrifuged at 7835 g for 5 min and filtered through a 0.22-μm Durapore membrane (Merck Millipore; Darmstadt, Germany). IgG and IgA antibody titers against the FimH, CsgA, PapG, FC, and FCP proteins were determined by ELISA using serum and urine samples diluted 1:50 and 1:10, respectively.

### Generation of polyclonal rabbit antibodies

Five female 6-month-old New Zealand rabbits were obtained from the “Instituto de Fisiología Celular de la Universidad Nacional Autónoma de México” for use in this study. Initially, the rabbits were immunized subcutaneously with 200 μg of FimH, CsgA, PapG, FC, and FCP proteins in complete Freund's adjuvant. The rabbits were re-immunized three times (days 21, 28, and 37) with 100 μg of each protein in incomplete Freund's adjuvant and bled via cardiac puncture on day 40. The collected blood was centrifuged at 7835 g for 5 min, and separated sera were stored at −70°C until use. The sera were absorbed using the CFT073 *csgA*::*km* + *fimH*::*cm* mutant strain generated in this study via a one-step inactivation method (Datsenko and Wanner, 2000). Anti-PapG and anti-FCP polyclonal rabbit sera were adsorbed using the CFT073 *csgA*::*km* + *fimH*::*cm* mutant strain under conditions that did not permit PapG expression, as confirmed by RT-PCR. Additionally, sera were heat-inactivated at 56°C for 30 min and titrated by ELISA using serial dilutions from 1:10 to 1:100,000 against the appropriate proteins.

### Adherence inhibition assay with HTB5 bladder cells

HTB5 bladder cells were cultured in 24 well-plates at a density of 10^5^ cells/well in 1 mL of fresh EMEM supplemented with 10% FBS until 80% confluence was reached. Previously, the strain CFT073 was grown overnight in LB broth at 37°C, and an aliquot (1:100 dilution) of the culture was incubated until it reached an OD_600_ of 1.0. The bacterial culture was then mixed with pre-immune or/and immune serum at a 1:1 ratio and maintained 2 h at 37°C with constant agitation. Cell monolayers were infected with 10^7^ bacterial cells [multiplicity of infection (MOI): 100] and incubated for 2 h at 37°C in 5% CO_2_. Infected cell monolayers were washed three times with PBS, and 200 μL of PBS containing 0.1% Triton X-100 was added. The bacteria that adhered to the cell monolayers were homogenized, and colony-forming units were determined using the Spotting Method described by Hannan and Hunstad (2016).

### Statistical analysis

One-way ANOVA and Student's *t*-test were used to compare differences between the mean and median values of groups using GraphPad Prism software (version 6). A *p* < 0.05 was considered statistically significant for all results.

## Results

### Structural components of the fusion proteins

Fusion proteins were generated using the sequences of the *fimH, papG*, and *csgA* genes, which exhibit identity of ≥90% among clinical UPEC strains. In addition, a signal peptide was identified between residues 1 and 27 of the sequences of the FimH and PapG proteins and residues 1–21 of the CsgA protein. Additionally, the CsgA protein contained a nucleation region between residues 22 and 41. These signal peptides and the nucleation region were eliminated from the sequences used to design and synthesize of the fusion proteins. The fusion of the FimH, PapG, and CsgA proteins was generated by employing the five repeats of the EAAAK linker ([EAAAK]_5_), which forms an alpha helix with a length of 39.95 Ȧ stabilized by five salt bridges between the Glu^−^ and Lys^+^ residues.

### Generation of dimeric and trimeric genes

**Figure 1:**
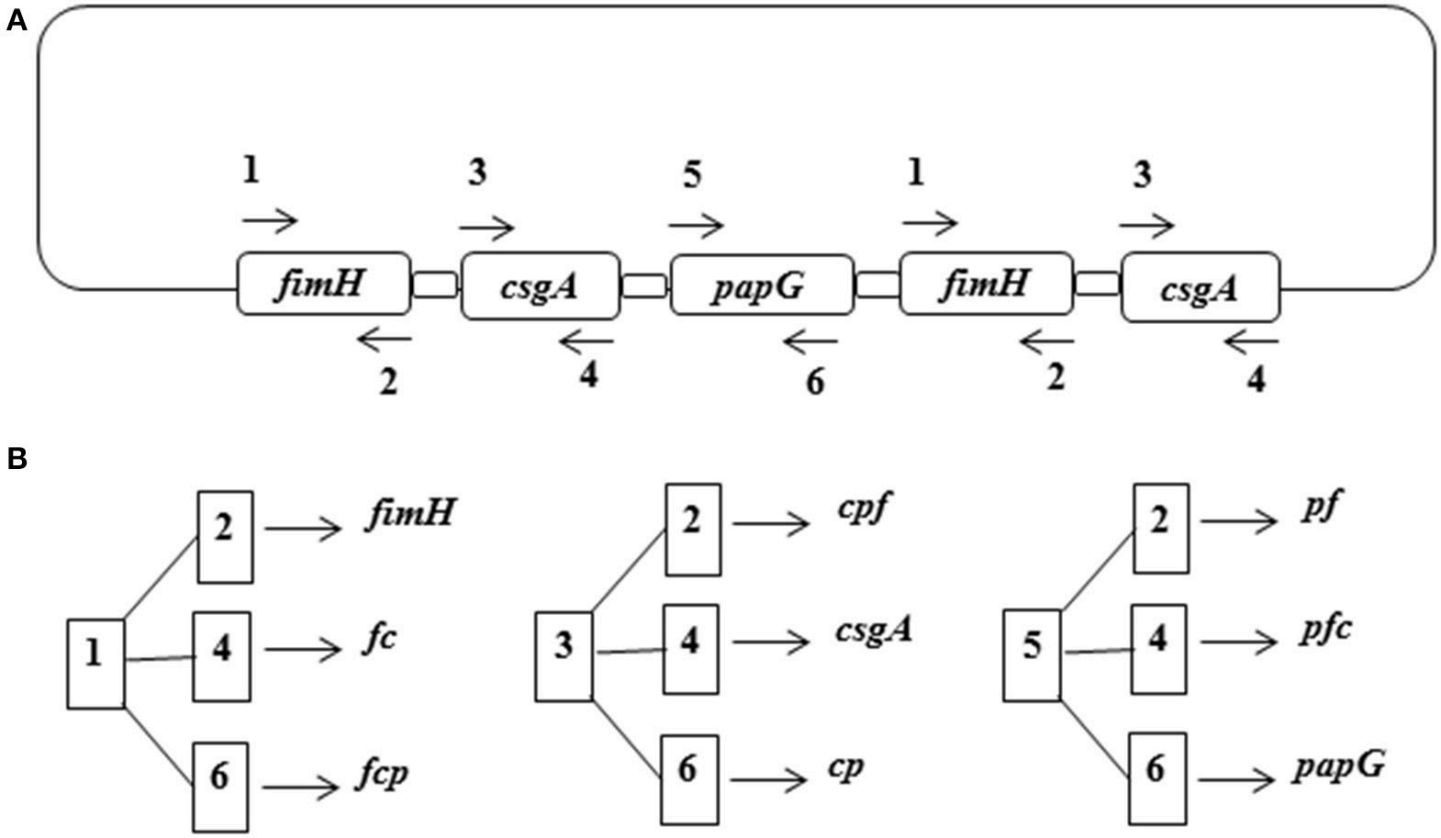
**Cloning strategy for the fusion proteins. (A)** The template gene sequence was constructed with the following order: *fimH*-*csgA*-*papG*-*fimH*-*csgA* linked with a GAAGCGGC GGCGAAA sequence repeated five times. **(B)** Generation of the fusion genes from the combination of specific primers for each gene. Dimeric and trimeric genes are abbreviated with the first letter of the *fimH* (*f*), *csgA* (*c*), and *papG* (*p*) genes.

A template fusion gene containing (in order) *fimH*-*csgA*-*papG*-*fimH*-*csgA* (*fcpfc*) linked to the nucleotide sequence of the [EAAAK]_5_ peptide was designed (Figure 1A). Six specific primers localized in the 5′ (forward) and 3′ (reverse) regions of each gene were designed and synthesized as described in the aLICator LIC Cloning and Expression Handbook (Table 1). Monomeric, dimeric, and trimeric genes were generated using combinations of the six primers in PCR amplification and cloned into the pLATE31 expression plasmid (Figure 1B).

**Table 1:** **List of primers used in this study**.

**Number**	**Primer**	**Sequence (5′-3′)**	**References**
1	FimH F	AGAAGGAGATATAACTATGAAAACCGCGAACGGTACCGCGATCCCGATCGGTGGTGGT	This study
2	FimH R	GTGGTGGTGATGGTGATGGCCCTGGTAAACGAAGGTAACACCGATGATAGACTGAAC	This study
3	CsgA F	AGAAGGAGATATAACTATGTCTGAACTGAACATCTACCAGTACGGTGGTGGTA	This study
4	CsgA R	GTGGTGGTGATGGTGATGGCCGTACTGGTGCGCGGTCGCGTTGTTACCGAA	This study
5	PapG F	AGAAGGAGATATAACTATGTCTCTGGGTAACGTTAACTCTTACCAGGGTGGTAA	This study
6	PapG R	GTGGTGGTGATGGTGATGGCCCGGCAGGATCATCAGCAGGGTCGCAGAACCAG	This study

### Synthesis of the template fusion gene

Increased expression levels of the fusion genes were obtained by preferential codon optimization of the *fcpfc* template fusion gene, which exhibited a CAI of 0.433–1.0 and a GC content of 48.9–55.4%. The *fcpfc*-optimized gene was synthesized chemically and cloned into the pUC57 vector to produce pF_2_C_2_P. Sequencing and digestion of the pF_2_C_2_P were consistent with the theoretical profile (data not shown).

### Primary and secondary structures

**Figure 2:**
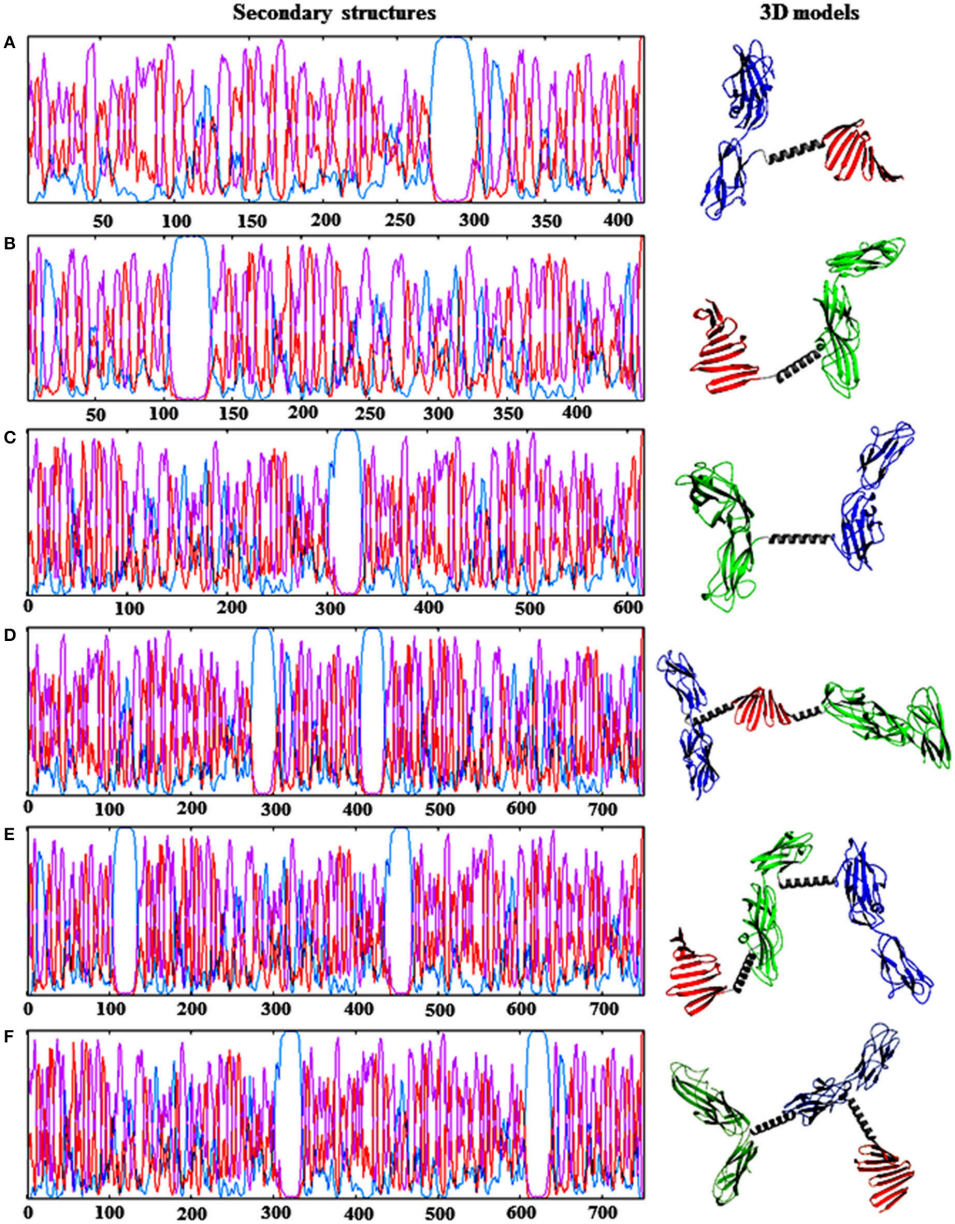
**Prediction of the secondary and tertiary structures of the fusion proteins**. **(A)** FC protein. **(B)** CP protein. **(C)** PF protein. **(D)** FCP protein. **(E)** CPF protein. **(F)** PFC protein. Dimeric and trimeric proteins were abbreviated (color) as follows: FimH (blue), CsgA (red), and PapG (green) proteins. Underlined and bold sequences correspond to the EAAAK linker (gray). Secondary structure prediction: (purple) extended strand, (red) coil, and (blue) helix.

The physical and chemical parameters of the monomeric (FimH, CsgA, and PapG), dimeric [FimH-CsgA (FC), CsgA-PapG (CP), and PapG-FimH (PF)], and trimeric [FimH-PapG-CsgA (FCP), PapG-CsgA-FimH (PCF), and CsgA-FimH-PapG (CFP)] proteins are shown in Table 2. Primary sequence analysis of these proteins revealed a high number of linear epitopes, between 18 and 33, as well as a high number of peptides binding to MHC-II, between 68 and 159 (Table 3). The secondary structure predictions for the monomeric, dimeric, and trimeric proteins indicated a high percentage of random coil and beta-sheet structures; however, lower percentages of alpha helices were observed (Figure 2). The conserved region of the alpha helix structure conferred by the [EAAAK]_5_ linker was identified in all fusion proteins.

**Table 2:** **Analysis of the physiochemical parameters of the fusion proteins**.

**Protein**	**No. of amino acids**	**Molecular weight (kDa)**	**Theoretical pI**	**Extinction coefficient**	**Estimated half-life**	**Instability index**	**Aliphatic index**	**GRAVY**
FimH	283	29.64	6.63	1.179	10	25.59	85.72	0.029
CsgA	116	12.06	5.58	0.826	10	16.23	51.38	−0.791
PapG	316	35.38	8.89	1.882	10	25.89	74.05	−0.364
FC	417	43.09	5.66	1.043	10	21.74	76.07	−0.183
CP	450	48.83	6.92	1.563	10	21.37	68.58	−0.443
PF	617	66.4	8.44	1.529	10	25.58	79.68	−0.161
FCP	751	79.85	6.74	1.397	10	22.37	75.39	−0.245
CPF	751	79.85	6.74	1.397	10	22.37	75.39	−0.245
PFC	751	79.85	6.74	1.397	10	22.37	75.39	−0.245

**Table 3:** **B-cell epitopes and MHC class II binding peptide predictions for the fusion proteins**.

**Fusion protein**	**Lineal epitopes**	**Conformational epitopes**	**MHC class II binding peptide**
FC	18	13	86
CP	27	12	68
PF	32	8	147
FCP	32	17	149
CPF	33	14	152
PFC	33	13	159

### 3D structures of the fusion proteins

The 3D structure models of the dimeric and trimeric proteins were refined 10 times to obtained the optimal minimization energy (Figure 2 and Table 4). Interestingly, the FC and FCP proteins exhibited models with optimal theoretical data that indicated a stable structural conformation. Gibbs free energy (ΔG) data indicated high stability for the FC protein, with ΔG = −7040.78 kcal/mol, and the FCP protein, with ΔG = −13,343.83 kcal/mol (Table 4). The 3D model validation of the FC and FCP proteins indicated that 94.7 and 93.1% of the amino acid residues were within permitted regions in the Ramachandran plot, with *Z*-scores of −6.95 (FC) and −9.53 (FCP).

**Table 4:** **Parameters for 3D modeling, refinement, and validation of the fusion proteins**.

**Fusion protein**	**Refinement cycle**	**Minimization energy (ΔG) (kcal/mol)**	**Ramachandran plot (%)**	***Z*-score**	**RMSD (FimH-PapG)**
FC	10	−11,912.82	94.70	−6.97	1.81 Ȧ−NA
CP	10	−7040.78	92.30	−7.7	NA−2.02 Ȧ
PF	10	−7140.54	92.40	−7.7	1.83 Ȧ−1.37 Ȧ
FCP	10	−13,866.68	93.10	−9.53	1.67 Ȧ−1.62 Ȧ
CPF	10	−13,343.83	92.50	−10.72	1.99 Ȧ−2.02 Ȧ
PFC	10	−13,577.65	91.90	−10.68	1.88 Ȧ−1.38 Ȧ

*Calculations for RMSD were generated with the FimH-lectin domain (PDB 1TR7) and PapG-lectin domain (PDB 1J8R). NA, not applicable*.

The 3D models of the FC and FCP proteins exhibited RMSDs of 1.81 and 1.67 Å, respectively, indicating conformations very similar to that of the mannose-binding domain of FimH. The FCP protein also exhibited an RMSD of 1.62 Å with the lectin domain of PapG (Table 4). Analysis of the 3D models using the Discotope program revealed 13 and 17 conformational epitopes in the FC and FCP proteins, respectively (Table 3).

### Generation of fusion proteins

**Figure 3:**
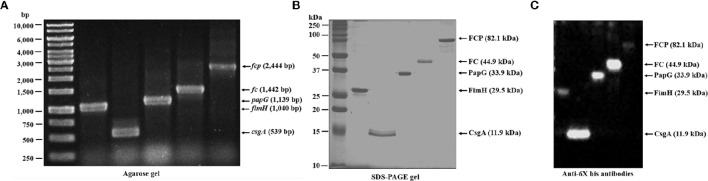
**Generation of the fusion proteins. (A)** The specific primers were combined to amplify the fusion genes by PCR, cloned in the pLATE31 plasmid and verified by colony PCR. **(B)** Fusion genes cloned into pLATE31 were used to transform BL21 (DE3) *E. coli*, followed by induction with 1 mM IPTG and purification of the 6His-tagged fusion proteins by Ni-NTA affinity chromatography. **(C)** Western blot assays with anti-6His (C-Term) HRP antibodies were employed to verify the fusion. Line 1 corresponds to FimH (1040 bp and 29.5 kDa), line 2 to CsgA (539 bp and 11.9 kDa), line 3 to PapG (1139 bp and 33.9 kDa), line 4 to FC (1442 bp and 44.9 kDa), and line 5 to FCP (2444 bp and 82.1 kDa). MW, molecular weight. bp, base pairs.

Bioinformatic analysis was an essential tool for the selection of monomeric (*fimH, csgA*, and *papG*), dimeric (*fc*), and trimeric (*fcp*) genes, which were cloned in the pLATE31 expression vector. PCR assays were performed to verify the cloned expression vectors using specific sequencing primers as described in the aLICator LIC Cloning and Expression Handbook (Figure 3A). The sequences of the fusion genes were determined by sequencing and will be submitted to the GenBank database (unpublished data). The BL21 (DE3) *E. coli* strain carrying the expression vectors was induced with 1 mM IPTG, and the His-tagged fusion proteins were purified under denaturing conditions by affinity chromatography. Coomassie blue-stained SDS-PAGE gels and Western blot analysis revealed 26.5, 11.9, 33.9, 44.9, and 82.1 kDa bands corresponding to FimH, CsgA, PapG, FC, and FCP, respectively (Figures 3B,C). The protein concentrations of FimH, CsgA, PapG, FC, and FCP were 2.7, 0.697, 2.63, 1.03, 0.998 mg/mL, respectively.

Mass spectrometry analysis by MALDI-TOF/TOF revealed the presence of specific peptides corresponding to the fusion proteins, and DLS analysis of the fusion proteins indicated a polydispersed state (Table 5).

**Table 5:** **Characterization of fusion proteins by DLS**.

**Sample name**	**T (°C)**	***Z*-Ave (d.nm)**	**PdI**
FimH	24.9	132.8	0.276
CsgA	24.9	2.97E + 04	0.948
PapG	25	264.9	0.253
FC	24.9	381.2	0.665
FCP	25	113.8	0.451

*PdI ≤ 0.04, monodispersed; PdI ≥ 0.04, polydispersed*.

### Induction of proinflammatory cytokine release by the fusion proteins

**Figure 4:**
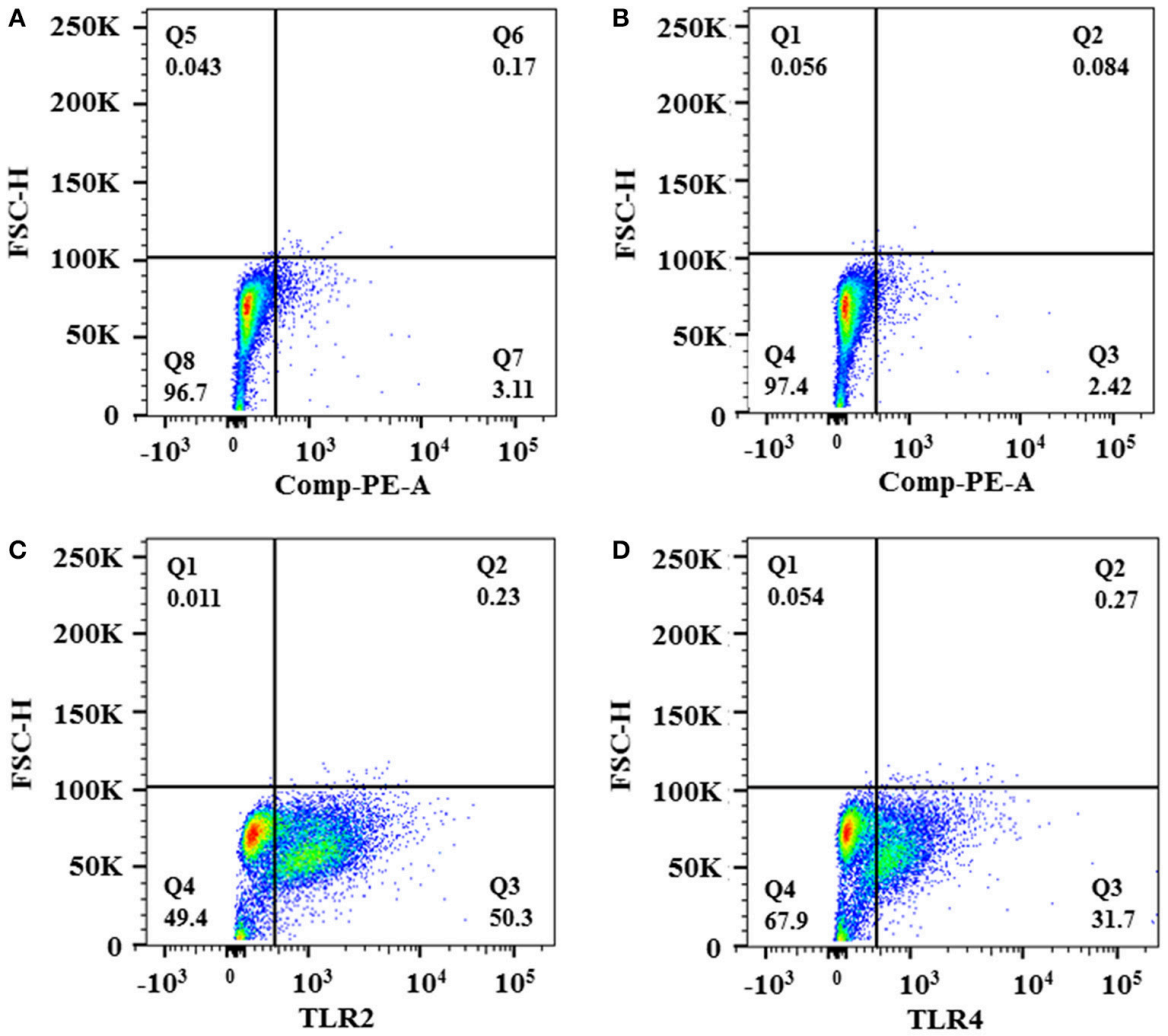
**Expression of TLR2 and TLR4 in HTB5 bladder cells**. Expression of TLR2 and TLR4 in HTB5 bladder cells was analyzed by flow cytometry. **(A)** Unstained HBT5 cells. **(B)** HTB5 cells incubated with an isotype control. **(C)** TLR2 expression in HTB5 cells analyzed using human TLR2 fluorescein-conjugated antibodies. **(D)** TLR4 expression in HTB5 cells analyzed using human TLR4/MD-2 complex phycoerythrin-conjugated antibodies.

**Figure 5:**
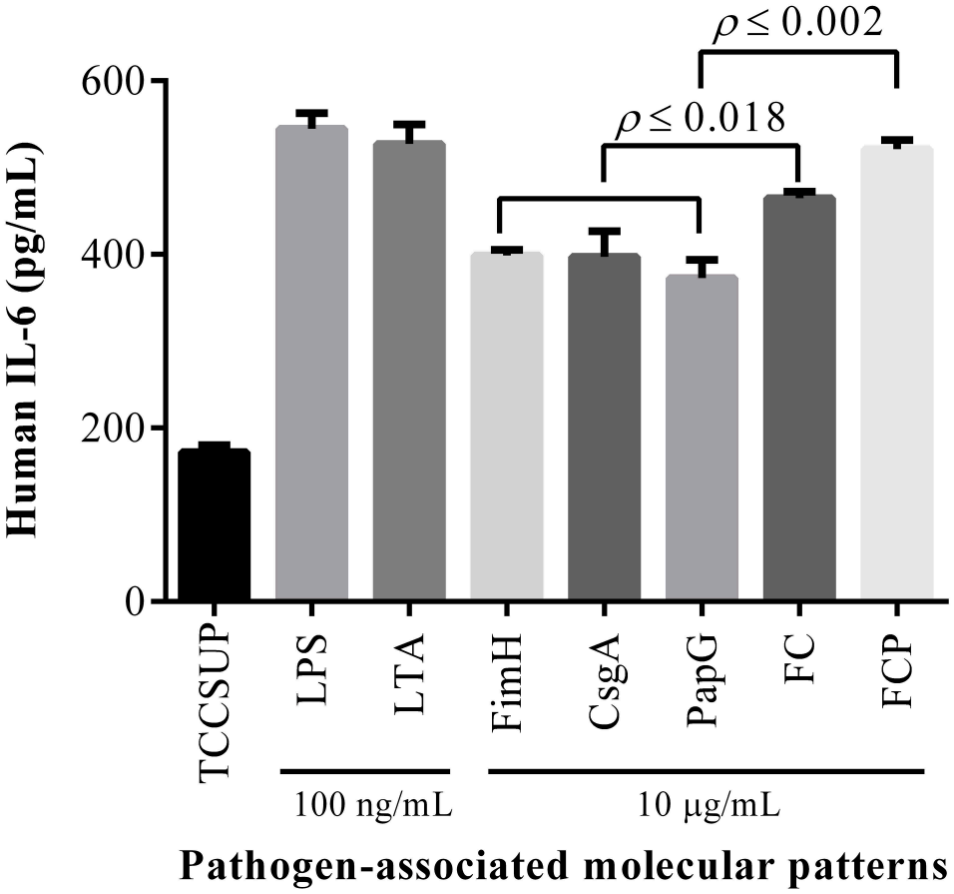
**Fusion proteins activate IL-6 release**. HTB5 cells were treated with 10 μg/mL of each fusion protein, and IL-6 release into the supernatant was detected by ELISA. Maximal induction of IL-6 was generated by the FCP protein, which exhibited significant (*p* ≤ 0.002) differences compared to the FC, FimH, CsgA, and PapG proteins. The FC protein also produced a significant increase (*p* ≤ 0.018) in IL-6 release compared to the FimH, CsgA, and PapG proteins. The bars represent the mean ± *S.D*. of three independent experiments. LPS and LTA (100 ng/mL) were used as controls.

**Figure 6:**
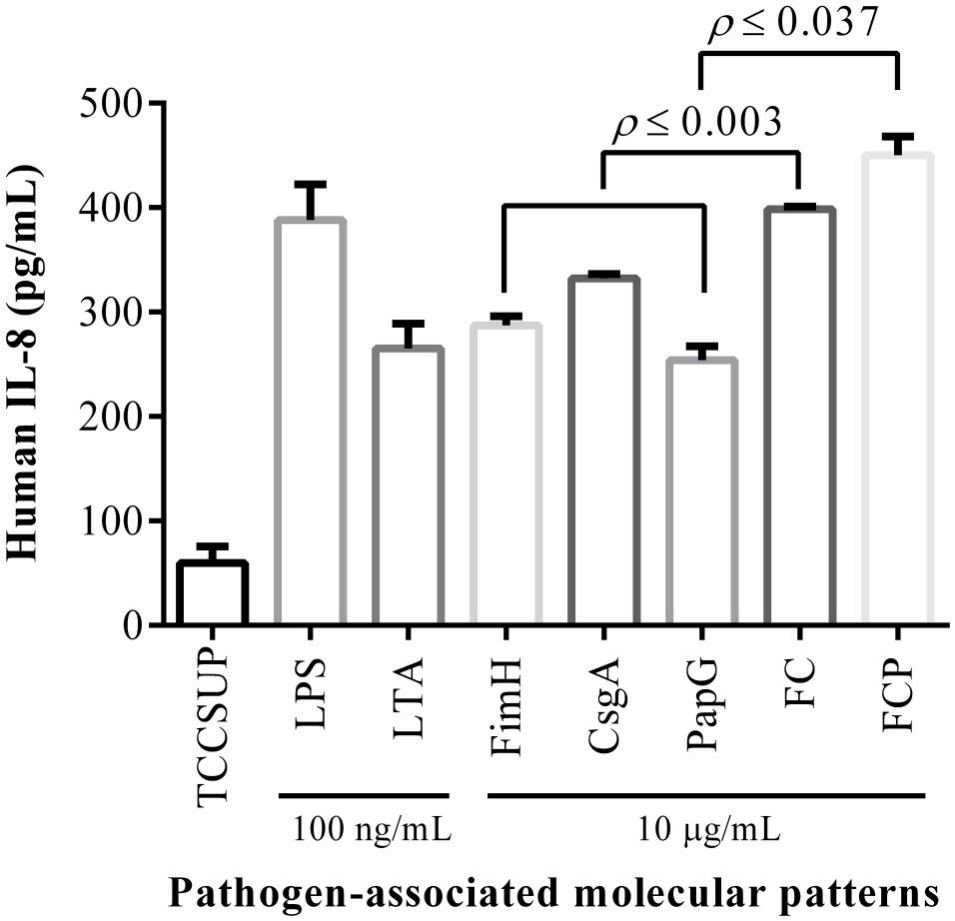
**The fusion proteins activate IL-8 release**. HTB5 cells were treated with 10 μg/mL of each fusion protein, and IL-8 release into the supernatant was detected by ELISA. A significant increase (*p* ≤ 0.037) in IL-8 release was induced by the FCP protein compared to the FC, FimH, CsgA, and PapG proteins. The FC protein also produced a significant increase (*p* ≤ 0.003) in IL-8 release compared to the FimH, CsgA, and PapG proteins. The bars represent the mean ± *S.D*. of three independent experiments. LPS and LTA (100 ng/mL) were used as controls.

Flow cytometry analysis of HTB5 bladder cells revealed an expression of 50.3% for TLR2 and 31.7% for TLR4 (Figure 4). Endotoxin levels ≤ 0.012 EU/mL were observed when the FimH, CsgA, PapG, FC, and FCP proteins were treated with 50 μg/mL polymyxin B. HTB5 bladder cells expressing TLR2 and TLR4 were incubated with 10 μg/mL FimH, CsgA, PapG, FC, or FCP, which induced different percentages of IL-6 and IL-8 cytokine release. Briefly, the FimH, CsgA, and PapG proteins stimulated the release of 372–398 pg/mL IL-6, and no significant differences in IL-6 release were observed among these proteins. However, the FC and FCP proteins induced the release of 464.79 and 521.24 pg/mL IL-6, respectively, which were significantly different (*p* ≤ 0.018 and *p* ≤ 0.002, respectively) from the values obtained for the monomeric proteins (Figure 5). In addition, 398.52 pg/mL IL-8 was released by the FC protein (*p* ≤ 0.003 compared to the monomeric protein), and 450.40 pg/mL IL-8 was released by the FCP protein (*p* ≤ 0.037 compared to the monomeric protein; Figure 6).

### Antigenicity of the fusion proteins

**Figure 7:**
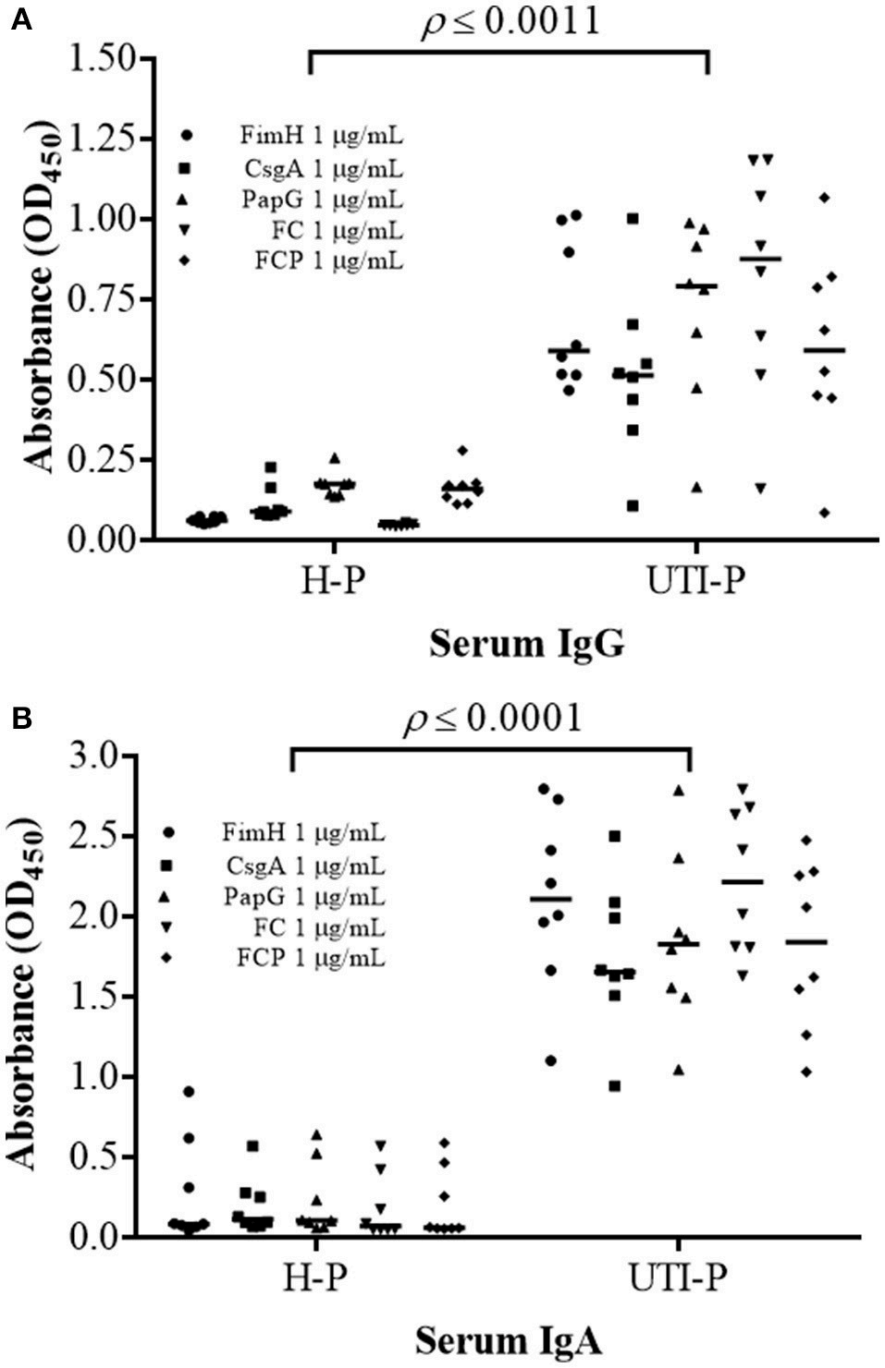
**Detection of IgG and IgA antibodies in the sera of patients with UTIs**. **(A)** A significant increase (*p* ≤ 0.0011) in IgG antibodies in UH-sera was detected compared to the values for IgG antibodies in HP-sera. **(B)** IgA antibodies in UP-sera were significantly increased (*p* ≤ 0.0001) compared with IgA antibodies in HP-sera. ELISA was performed in triplicate using three different samples, and 1 μg/mL each protein was used. The points represent individuals, and the bars represent the median.

**Figure 8:**
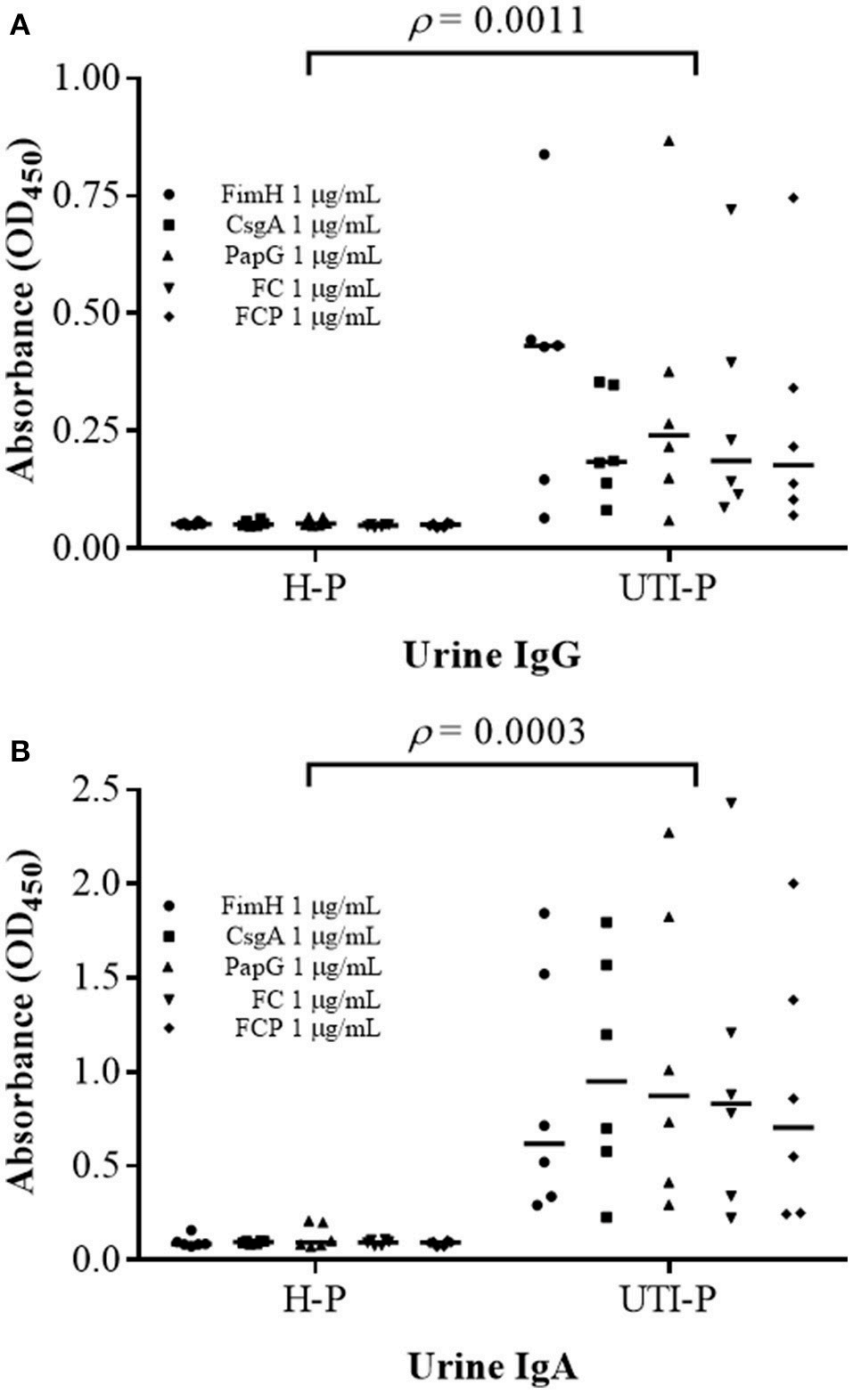
**Detection of IgG and IgA antibodies in the urine of patients with UTIs**. **(A)** A significant increase (*p* = 0.0011) in IgG antibodies in the urine of UP patients was detected compared to the values for IgG antibodies in HP-sera. **(B)** IgA antibodies in UP-urine were significantly increased (*p* = 0.0003) compared with IgA antibodies in HP-sera. ELISA was performed in triplicate using three different samples, and 1 μg/mL each protein was used. The points represent individuals, and the bars represent the median.

ELISA assays were performed to detect antibodies against the FimH, CsgA, PapG, FC, and FCP proteins in the sera and urine of HP and UP (Figures 7, 8). Antibodies in the sera reacted with all proteins evaluated. High levels of UP-IgA antibodies in sera were observed, with a median OD_450_ of 1.628–2.216. Interestingly, high levels of UP-IgA antibodies in sera, with a median OD_450_ of 2.216 were detected when ELISA was performed using the FC protein as an antigen (Figure 7B). However, under identical conditions, low levels of UP-IgG (median values of 0.514–0.837) were detected. The FC protein exhibited greater specificity, with sera detecting UP-IgG with a median value of 0.837 (Figure 7A). Additionally, all fusion proteins reacted with high levels of UP-IgA antibodies in urine, with a significant difference of *p* = 0.0003 compared to the values for HP-IgA antibodies. Furthermore, 1 μg/mL fusion protein detected high levels of UP-IgA antibodies in urine, with median values ranging from 0.6189 to 0.950 (Figure 8B). In contrast, low levels of UP-IgG were detected in urine, with median values ranging from 0.444 to 0.619 (Figure 8A). The difference in the values between UP-IgA and UP-IgG in urine was significant (*p* < 0.05) compared to HP-IgA and HP-IgG (Figure 8).

### Polyclonal rabbit antibodies block bacterial adherence

**Figure 9:**
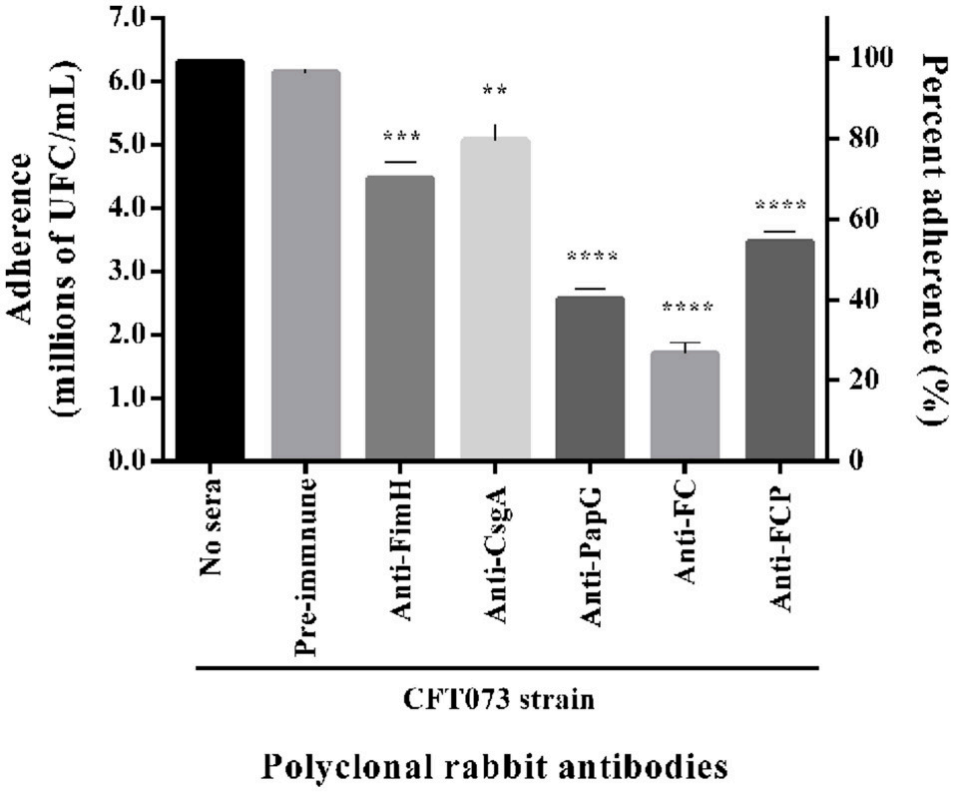
**Reduction in bacterial adherence in the presence of polyclonal rabbit antibodies**. *E. coli* strain CFT073 was incubated with 50% sera from rabbits immunized with the FimH, CsgA, PapG, FC, and FCP proteins and HTB5 bladder cells (MOI 1:100) for 2 h. Polyclonal rabbit antibodies favored differing reductions in bacterial adherence (CFU/mL and percentage) compared with basal adherence of the strain CFT073 and pooled pre-immune sera. The bars represent the mean ± *S.D*. of three independent experiments. **p = 0.0011, ***p = 0.0002, and ****p < 0.0001.

Polyclonal rabbit antibodies against the FimH, CsgA, PapG, FC, and FCP proteins were used to block bacterial adherence to HTB5 bladder cells. The strain CFT073 showed adherence to 6.3 × 10^6^ HTB5 bladder cells, and this level of adherence was set at 100%. Similar adherence by the strain CFT073 was observed with pooled pre-immune sera (Figure 9). Bacterial adherence in the presence of polyclonal rabbit antibodies showed a significant reduction to 32% for anti-FimH (*p* = 0.002), 21% for anti-CsgA (*p* = 0.0011), 60% for anti-PapG (*p* < 0.0001), 73% for anti-FC (*p* < 0.0001), and 46% for anti-FCP (*p* < 0.0001) compared with the CFT073 and pre-immune serum controls (Figure 9).

## Discussion

UTIs are the second most common infection in humans and are associated with high annual economic costs due to patient morbidity. UPEC is the most common etiologic agent of UTIs (Foxman, 2014). Increased antibiotic resistance among UPEC strains has reduced treatment options and significantly increased UTI-associated morbidity and mortality (Melzer and Petersen, 2007; Molina-López et al., 2011). Research to date has focused on more effective and less costly options for antibiotic therapy to treat UTIs and vaccination as a viable alternative due to increasing resistance among UPEC strains (Serino et al., 2010; Sivick and Mobley, 2010; Brumbaugh and Mobley, 2012; Moriel and Schembri, 2013; Mobley and Alteri, 2015).

Fusion technology has been utilized to design new vaccines due to its ability to induce a rapid cellular and humoral immunity response against UPEC (Huleatt et al., 2007). Fimbrial adhesin plays a critical role in UPEC binding, colonization, and bladder and kidney cell invasion (Spaulding and Hultgren, 2016). We hypothesized that blocking adhesion using anti-fimbrial adhesin antibodies would inhibit the initial step in the UPEC pathogenic mechanism. Fusion proteins comprising the FimH, PapG, and CsgA adhesins and EAAAK rigid linkers were designed and analyzed using bioinformatics tools. Based on bioinformatics, we selected the [EAAAK]_5_ peptide, which contains a longer helix that results in appropriate separation among fusion proteins. Small-angle X-ray scattering data have revealed that multimerization is caused by short helical linkers (*n* = 2–3), whereas longer linkers (*n* = 4–5) solvate monomeric fusion proteins (Arai et al., 2004; Zagrovic et al., 2005). The monomeric, dimeric, and trimeric fusion genes generated from the *fcpfc* optimized-template exhibited a CAI value of 1.0 and a high GC content (55.4%). Thus, the designed sequence appears to be optimal for expressing all fusion proteins, as described in previous studies (Brinkmann et al., 1989; Andrews et al., 1996; Pedersen-Lane et al., 1997). Three different variants of the dimeric (*fc, cp*, and *pf*) and trimeric (*fcp, cpf*, and *pfc*) genes were designed to construct and express the fusion proteins. However, based on theoretical analysis of the fusion proteins, FC, and FCP were selected for their structural stability and folding. *In silico* studies of fusion proteins based on FimH from UPEC and MrpH from *Proteus mirabilis* have revealed that the sequence order is critical for stability and folding (Habibi et al., 2015a). Bioinformatics tools were recently used to propose potential vaccine designs for *Helicobacter pylori*, enterotoxigenic *E. coli, Brucella*, influenza viruses, and UPEC (Nazarian et al., 2012; Savar et al., 2014; Golshani et al., 2015; Muñoz-Medina et al., 2015; Habibi et al., 2015b; Mohammad et al., 2016). Additionally, vaccines have been designed to contain T-cell and B-cell immune epitopes, and a class II MHC peptide was also identified using bioinformatics tools (De Groot et al., 2002; De Groot and Moise, 2007). Based on these studies, we evaluated the physicochemical properties of the amino acid residues, linear epitopes and peptides binding MHC-II in the primary sequences of the fusion proteins. Prediction of the physicochemical properties of the fusion proteins by our “*in silico*” analysis suggested high amounts of epitopes and peptides that bound MHC-II; furthermore, there appeared to be a relationship between the number of epitopes and the physicochemical properties of the fusion proteins (Sim et al., 1996; Huang et al., 2016).

The FimH protein contains a mannose-binding lectin domain between residues 1 and 150 and a pilin domain between residues 159 and 279, with eight amino acids connecting the two domains (Hung et al., 2002). We identified epitopes and peptides in the minimal mannose-binding region of FimH (residues 1–25), a region that has been reported to confer protection against UTIs caused by UPEC (Thankavel et al., 1997). Similarly, the PapG protein contains a lectin domain between residues 1 and 198 and a pilin domain between residues 206 and 336 (Sung et al., 2001). Our data revealed the presence of both epitopes and peptides of the PapG protein in its Gal(α1-4)Gal-binding region, between residues 1 and 63 and residues 155 and 173 (Sung et al., 2001). Moreover, CsgA protein prediction revealed two epitopes localized in repetitions 4 and 5 that are protein-binding, functional regions characterized by several beta-turn structures and exposed domains exhibiting a hydrophilic nature in other amyloid proteins (Ikai, 1980; Olsén et al., 2002). Interestingly, the [EAAAK]_5_ linker used in the fusion proteins does not contain B-cell epitopes and MHC class II-binding peptides. Moreover, the fusion proteins exhibited a high percentage of random coil structure, which have been reported in other models as principal components of antigens with flexible regions that allow protein-protein interactions (Janin and Chothia, 1990; Chen et al., 2007).

3D structure prediction of the fusion proteins confirmed their stability and correct conformation based on refinement, energy minimization, and validation analyses. An RMSD value of 1–2 Å was calculated based on the superimposition of low-resolution X-ray structures, thus indicating that the fusion proteins maintained their structures and revealing the mannose-binding and lectin domains of FimH and PapG in the fusion proteins. Remarkably, stability and structure are properties that are related to protein function. In addition, the CsgA protein has not yet been resolved by crystallography, though circular dichroism, X-ray diffraction, and NMR data for Curli fibers suggest the presence of parallel β-sheet structures. 3D models of CsgA, dimeric (FC and CP), and trimeric proteins proposed in this study reveal the presence of parallel β-sheet structures (Shewmaker et al., 2009). Interestingly, the FC and FCP proteins exhibited high stability and a greater number of epitopes than the other fusion proteins. FimH, CsgA, and PapG were used as controls and compared to the fusion proteins. Theoretical data obtained by bioinformatics tools reduced the risk of failure of the experimental approaches and optimized resources for generating on the FC and FCP proteins (Luscombe et al., 2001).

The FimH, CsgA, PapG, FC, and FCP proteins in inclusion bodies were purified under denaturing conditions. Several proteins in inclusion bodies have been purified using chaotropic agents, such as urea and GdnHCl (Wang et al., 2007; Seras-Franzoso et al., 2015). Purified CsgA protein was visualized on SDS-PAGE gels only after formic acid treatment, indicating the formation of amyloid functional aggregates. DLS analysis revealed a polydispersity index (PdI) of 0.948 and a *Z*-Average size of 2.97 × 10^4^ nm, which confirmed the aggregation rate of the CsgA-CsgA interaction. The PdI and Z-Average values of the FC protein were greater than those of the FCP protein, which indicates that CsgA linked to FimH and/or PapG promotes low aggregate formation by the fusion proteins because the domains of the CsgA proteins were not exposed to interactions with other CsgA monomers. Therefore, these fusion proteins do not require pretreatment with formic acid for visualization in SDS-PAGE gels.

Fusion proteins with bioactivity that depends on separation (linker), stability, and folding have been described (Chen et al., 2013). Fimbriae are involved in cytokine release by pathogen-associated molecular patterns (PAMPs), which are capable of recognizing pattern recognition receptors (PRRs) such as TLR4 in the mucosa of the urinary tract (Sirard et al., 2006). We suggest a dependence of the fusion protein-PPR interaction with regard to the correct structural conformation to induce a pro-inflammatory response, as described in other models (Akira et al., 2001). A recent study demonstrated that Curli fibers (CsgA) bind to the TLR2-TLR1 dimer to produce IL-6 release from marrow-derived macrophages (Rapsinski et al., 2015). Based on these results, the presence of TLR2 and TLR4 in HTB5 bladder cells from anaplastic transitional cell carcinoma was described for the first time in this study (Nayak et al., 1977). In addition, our data showed that the FimH and PapG proteins could induce release of IL-6 and IL-8, which bind specifically to TLR4 present on immune cells and the surface of uroepithelial cells (Frendéus et al., 2001; Fischer et al., 2006). Compared to the values obtained for FimH, CsgA, and PapG, the FC and FCP proteins generated in this study induced significant increases in IL-6 and IL-8 release. Our data showed that the functionality of the fusion proteins is involved in the release of high levels of IL-6 and IL-8 when compared to monomeric proteins, probably due to the synergistic signaling of TLR2 and TLR4, an example UPEC strains which have been linked to the activation of TLR4 and TLR5 pathways, thereby initiating innate immune responses against UTIs (Cheng et al., 2016). Conversely, fusion proteins may be considered PAMPs that are capable of recognizing PRRs as proteins with adjuvant-type characteristics (Huleatt et al., 2007).

Potential antigens for ideal vaccines should have features such as surface exposure, expression during infection and immunogenic properties (Sivick and Mobley, 2010; Brumbaugh and Mobley, 2012; Mobley and Alteri, 2015). FimH, CsgA, and PapG proteins are adhesins located on the UPEC surface (Antão et al., 2009). An immunoproteomic approach has been used to identify target antigens and viable targets for vaccine development (Hagan and Mobley, 2007). An immunoproteomic test based on ELISA analysis of sera and urine from UTI patients using the fusion proteins was used to determine the antigenicity and *in vivo* expression of FimH, PapG, and CsgA. Using the FimH, CsgA, PapG, FC, and FCP proteins, higher levels of UP-IgA antibodies were detected in both sera and urine compared to HP-IgA and UP-IgG antibodies. These data are consistent with studies using other UPEC proteins (Rene et al., 1982; Rene and Silverblatt, 1982). Additionally, the antibodies detected in serum and urine also confirmed FimH, CsgA, and PapG protein expression *in vivo* during UTIs caused by UPEC. FC protein contains exposed epitopes that were recognized by antibodies in sera and urine, as confirmed by bioinformatics analysis. However, less robust antigenicity was observed for FCP than FC protein, likely due to the structural complexity of the former, which hides potential epitopes. The titration curves for polyclonal rabbit antibodies against the FCP protein revealed titers > 1:100,000; however, the anti-CsgA antibodies had titers < 1:1000, indicating that CsgA may be hidden by 3D folding of the FimH and PapG proteins (data not shown). A significant reduction in bacterial adherence by strain CFT073 to HTB5 bladder cells was observed with the anti-PapG, anti-FC, and anti-FCP polyclonal rabbit antibodies compared to pre-immune sera and without antibodies.

In conclusion, recombinant fusion proteins designated FC and FCP were designed and selected for bioinformatics analysis and were observed to significantly induce the release of IL-6 and IL-8. In addition, these proteins exhibited antigenicity based on the detection of IgG and IgA antibodies in the sera and urine of UTI patients and the protective functions of antibodies targeting the fusion proteins *in vitro*. These data suggest that FC and FCP proteins are potential biomolecules for generating vaccines against UTIs caused by UPEC. However, immunogenicity studies of the FC and FCP proteins are needed to achieve the goal of developing a vaccine.

## Author contributions

Designed and conceived the experiments: VL, JR, and JX. Performed the experiments: VL, ZS, CM, and VC. Analyzed the data: VL, AC, LM, and JX. Contributed reagents/materials/analysis tools: SO, AC, JA, RH, and JX. Wrote and reviewed the manuscript: VL and JX.

## Funding

Public Federal Funds grant number HIM/2014/022 and HIM/2016/027 from the HIMFG supported this work.

### Conflict of interest statement

The authors declare that the research was conducted in the absence of any commercial or financial relationships that could be construed as a potential conflict of interest.
